# Supplementary figures and images for: The role of ecological niche evolution on diversification patterns of birds distinctly distributed between the Amazonia and Atlantic rainforests
Source: PLoS One. 2020 Oct 13;15(10):e0238729. doi: 10.1371/journal.pone.0238729 (PMC7553277; doi:10.1371/journal.pone.0238729)

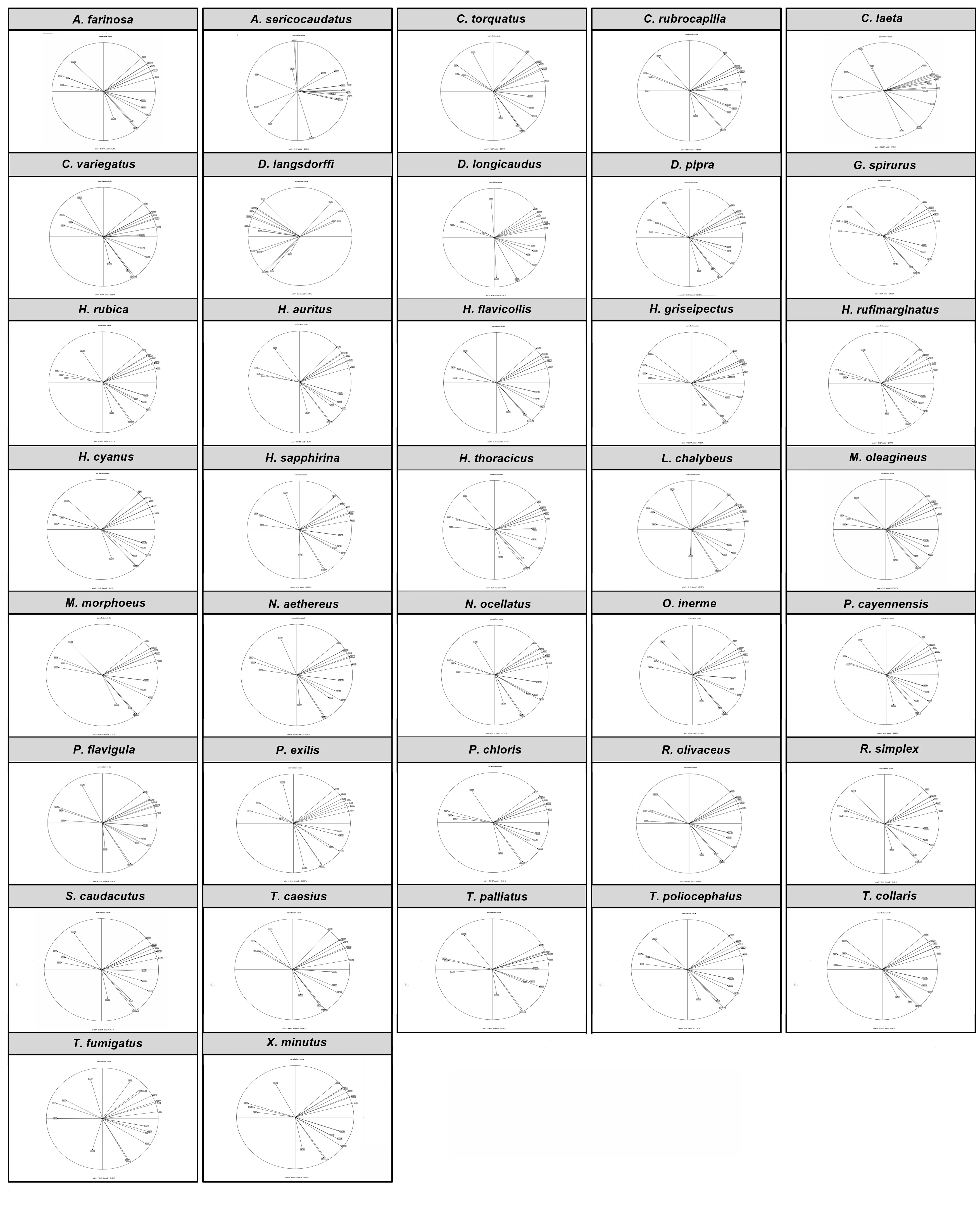

Supplement: S1 Fig — (TIF) [file pone.0238729.s003.tif]
