# Supplementary material for: The role of ecological niche evolution on diversification patterns of birds distinctly distributed between the Amazonia and Atlantic rainforests
Source: PLoS One. 2020 Oct 13;15(10):e0238729. doi: 10.1371/journal.pone.0238729 (PMC7553277; doi:10.1371/journal.pone.0238729)
Supplement: Attachment — Submitted filename: Response to Reviewers.docx [file pone.0238729.s005.docx]

Response to Reviewer

Dear Editor Dr Stefan Lötters,

We thank you and reviewer for the useful comments and suggestions. We have carefully checked all the recommendations and have now revised and modified the text accordingly. We did all the minor changes to the manuscript as requested. Attached to this letter, we provide specific answers to each of the reviewer’s concern.

We appreciate all the positive feedback and the specific comments provided.

Sincerely,

Ricardo Ribeiro da Silva, M.D. (Corresponding author, on behalf of all authors)

**ANSWERS TO THE REVIEWERS**

**Reviewer:**

**Comments to the Author**

**Reviewer #1**

**Q#1: Authors made a very good job in reviewing the manuscript. This new version is substantially improved. I am satisfied with modifications and author’s responses. I have some minor comments:**

Thank you for the positive feedback, your comments were fundamental to guide our revision.

**TITLE: change the word "speciation" to "diversification" as most of isolated populations on both biomes are not ranked as distinct species.**

Q#1: We changed the word "speciation" to "diversification" in the title.

**Q#2: Lines 26-28: This statement was proposed by classical papers, instead these new ones. Please correct the citation including Haffer (1969) and Brown & Ab’Saber (1979).**

A#2: Thanks for pointing this out. We have corrected the citation including these articles.

**Q#3: Lines 194-197: Why do the authors choose these thresholds? It is better use commonly used values from previous studies, than arbitrary values.**

Q#3: We changed and standardized the limit value based on previous studies (Manel et al. 1999, 2001, Luck 2002, Stockwell and Peterson 2002, Bailey et al. 2002, Woolf et al. 2002, Liu 2005), considering the value of 0.05 in the lowest category to optimize the percentage of models.

**Q#4: Line 233: correct is 0.05.**

Q#4: We modified the text to the correct value.

**Q#5: Lines 247-248: Actually, your results indicate both niche divergence and conservatism. You should put in evidence both scenarios, as evidenced in your results.**

Q#5: We agree, the entire paragraph show that. But we moved the sentence up to make it clearer. The text now reads as follow:

“Our results indicate that bird populations that have disjunct distribution in the Amazon and Atlantic Forest show signs of Grinellian niche divergence, mainly supported by the low niche overlap among populations of the same species. Although, underlying processes of niche conservatism seemingly constrain niche evolution in these species because for nearly half of the studied species, observed niche overlap — although small — tended to be higher than what would just be expected by chance (similarity test results).”

**Q#6:** **Lines 257-258: what do you mean with "considered evolutionary time"? We might expect recent splits for most co-distributed species in both biomes, due to lack of phenotypic divergence.**

A#6 We were referring to the short evolutionary time. We changed the sentence to be more specific and avoid confusion. The sentence now reads as follow:

“Our results represent one of the few examples where niche divergence can occur under the such short evolutionary time.”

**Q#7:** **Line 259: which phylogeographic studies?**

Q#7: We updated the text to indicate the proper references of the phylogeographic studies.

**Q#8: Lines 301-302: include Ledo and Coli (2017) and Batalha-Filho et al (2013) in the citations, as they properly showed this hypothesis.**

Q#8: We added the appropriate citations.
